# Clinical Profiles of Neonates Born to COVID-19 Positive Mothers in a Tertiary Care Centre: A Descriptive Cross-sectional Study

**DOI:** 10.31729/jnma.6808

**Published:** 2021-07-31

**Authors:** Shiva Prasad Sharma Chalise, Santosh Kumar Mishra, Bimal Sharma Chalise, Punam Rai, Subash Paudel, Prerana Kansakar, Anil Raj Ojha

**Affiliations:** 1 Department of Pediatrics, Patan Academy of Health Sciences, Laiitpur, Nepal; 2 Sukraraj Tropical and Infectious Disease Hospital, Teku, Kathmandu, Nepal

**Keywords:** *COVID-19*, *mothers*, *neonate*

## Abstract

**Introduction:**

Nepal is one of the countries which has been hit hard by the COVID-19 pandemic and has resulted in high morbidity and mortality across all age groups including neonates. There has been a paucity of studies regarding maternal to neonatal COVID-19 transmission and the published studies also have a poor sample size to reach any definite conclusion. Hence this study was carried out to see the clinical profiles of neonates born to COVID-19 mothers.

**Methods:**

It was a descriptive cross-sectional study. The study was conducted at a tertiary care centre over the period of one year from April 2020 to March 2021 after taking ethical clearance from the Institutional Review Committee with reference number drs2105211526. Convenient sampling was done. All neonates born to COVID-19 positive mothers who were diagnosed by a real-time polymerase chain reaction of the nasopharyngeal swab during the time of delivery were included in the study. Data analysis was done using Statistical Package for Social Sciences 20 using appropriate tools.

**Results:**

A total of 105 babies born to COVID-19 positive mothers who were tested for COVID-19 infection were included in the study. Ten (9.5%) (3.89-15.10 at 95% Confidence Interval) of neonates born to COVID-19 positive mothers were positive for the COVID-19 virus. All the neonates born to COVID-19 positive mothers were discharged home except one case who had other comorbidities. Fever was present in four (40%) of COVID-19 positive neonates.

**Conclusions:**

There is a possibility of vertical transmission of coronavirus in neonates although the outcome is favourable.

## INTRODUCTION

Nepal is one of the countries which has been hit hard by COVID-19 pandemic and has resulted in high morbidity and mortality across all age group including neonates. It has also caused significant reduction in institutional delivery with a negative impact on quality care thus leading to increase in institutional stillbirth and neonatal mortality rate.^1^

Studies have shown that up to ten percent of neonates born to COVID-19 positive mothers have positive viral tests.^2^ However a study done in Southeast Asia showed no maternal to neonatal COVID-19 transmission.^3^ There has been paucity of studies regarding maternal to neonatal COVID-19 transmission and the published studies also have poor sample size to reach any definite conclusion.^2,3^

Hence this study was carried out to see the clinical profiles of neonates born to COVID-19 positive mothers in a tertiary care hospital in Nepal.

## METHODS

It was a descriptive cross-sectional study. The study was conducted in Patan Hospital, Lalitpur over the period of one year from April 2020 to March 2021. The study was conducted after taking ethical clearance from the Institutional Review Committee with reference number drs210521 1526. All neonates born to COVID-19 positive mothers who were diagnosed by real time polymerase chain reaction (RT PCR) of nasopharyngeal swab during the time of delivery were included in the study. Neonates born to antigen positive but RT PCR negative mothers, neonates born to COVID-19 positive mothers but not tested for COVID-19 and neonates whose file were missing were excluded from the study. Convenient sampling was done and the sample size was calculated using the formula,

n = Z^2^ × p × q / e^2^

  = (1.96)^2^ × 0.1 × (1-0.1) / (0.06)^2^

  = 96

Where,

n = minimum required sample size
Z = 1.96 at 95% Confidence Interval (CI)
p = prevalence from previous study 10%^2^
q = 1-p
e = margin of error, 6%

Though the convenient sampling was 96, we included all cases admitted in the time frame and collected data from 105 cases.

RT-PCR of all neonates born to COVID-19 positive mothers was sent at approximately 24 hours after delivery according to the hospital protocol. Maternal data of COVID-19 positive mother like age, pre-existing comorbidities like diabetes, hypertension, per vaginal leaking, gravida, parity, meconium-stained liquor, mode of delivery, antenatal care visits were recorded. Baby's data like gestational age, birth weight, Apgar score, COVID-19 status, transient tachypnoea of new born, respiratory distress syndrome, pneumonia, sepsis, need of mechanical ventilation, need of inotropes and duration of hospital stay were reviewed. Data were analysed using Statistical Package for Social Sciences (SPSS) version 20. Mean, median and standard deviation were used for continuous and normally distributed variables.

## RESULTS

There were 107 babies born to COVID-19 positive mothers. Two cases were excluded as RT PCR was not sent. Final analysis was done among 1 05 cases. Ten (9.5%) (3.89-15.10 at 95% Confidence Interval) neonates were positive for COVID-19 PCR. Among all babies born to COVID-19 positive mothers, 38 (36.2 %) had antenatal follow up outside the hospital. Mean and median maternal age was 28.4 and 28 with standard deviation of 4.4 years and range of 17-39 years. Fifty two percent were male and forty eight percent were female. Mean and median weight of the baby was 2928 and 2990 grams with standard deviation of 574 grams and range of 840-3970 grams. Among them ninety-two (87.6%) were term babies (Table 1 and Table 2).

**Table 1:** Maternal details of neonates born to COVID-19 positive mothers.

Maternal details	n (%)
Primigravida	49 (46.7)
Caesarean delivery	63 (60)
Gestational diabetes	4 (3.8)
Gestational hypertension	1 (0.95)
PV leaking >18 hours	2 (1.9)
Meconium-stained liquor	10 (9.5)

**Table 2:** Baby details of neonates born to COVID-19 positive mothers.

Baby details	n (%)
Gestational age < 37 weeks	13 (12.4)
Birth weight < 2500 grams	15 (14.3)
Apgar score at 1 minutes < 7	10 (9.5)
Need of resuscitation	4 (3.8)
COVID-19 Positive	10 (9.5)

**Figure 1:**
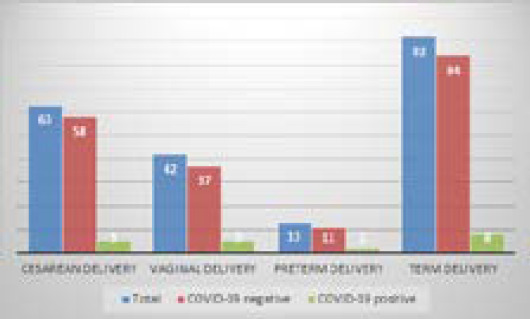
Mode of deliverity and maturity of neonates born to COVID-19 positive mothers.

Among them forty-two were delivered vaginally and sixty-three were delivered by caesarean section. Mode of delivery and maturity of the babies born to COVID-19 positive mothers (Figure 1).

Five among them had symptoms. Clinical profiles of neonates born to COVID-19 positive mothers has been given (Table 3). Fever which was present in four cases. Three patient required respiratory support, two for respiratory distress syndrome and one for meconium aspiration syndrome. One baby expired among all cases. The baby was PCR positive but had other severe comorbidities. The patient was born at 30 weeks of gestation and required active resuscitation at birth. The baby also had tracheoesophageal fistula and surgery was planned but not done due to hemodynamic instability. The baby required ventilator and inotropic support for 13 days. The baby expired at 13 days of life.

Among all cases eighty-one babies were discharged after PCR report and discussion with parents within 72 hours. Ninety-three patients were discharged within seven days and twelve patients had hospital stay of more than seven days. Among them eight were preterm, one was culture positive (coagulase negative staphylococcus) sepsis, one was birth asphyxia, another one treated as meningitis, one congenital heart disease. Four of them stayed for more than two weeks among them three were preterm and one was meningitis.

**Table 3:** Clinical profiles of neonates born to COVID-19 positive mothers.

Clinical profile	COVID-19 Positive neonates (n=10) (%)	COVID-19 Negative neonates (n=95) n (%)	All Neonates born to COVID-19 positive mothers (n=105) n (%)
Mechanical ventilation	2 (20)	3 (3.2)	5 (4.8)
Need of inotropes	1 (10)	1 (1.0)	2 (1.9)
Fever	4 (40)	9 (9.5)	13 (12.4)
Oxygen required	3 (30)	8 (8.4)	11 (10.5)
Transient tachypnea of newborn	0 (0)	5 (5.3)	5 (4.8)
Respiratory distress syndrome	2 (20)	3 (3.2)	5 (4.8)
Meconium aspiration syndrome	1 (10)	0 (0)	1 (1)
Sepsis (Coagulase negative staphylococcus)	0 (0)	1 (1.0)	1 (1)

## DISCUSSION

Centre for Disease Control and Prevention (CDC) recommends COVID-19 testing of all new-borns born to COVID-19 positive mothers at approximately 24 hours and 48 hours of life irrespective of sign and symptoms.^4^ There are conflicting results from previous studies on the risk of vertical transmission of coronavirus in neonates. This study shows 9.5 percent of neonates born to COVID-19 positive mother to be positive for COVID-19 virus. This result is similar to a study from India in which out of 65 tested neonates, 10.7% were confirmed COVID-19 positive by RTPCR.^2^ The study further showed viral loads of mothers with COVID-19 positive and negative neonates were comparable. In another study from India, among 221 new-borns 14.47 percent were positive.^5^ In another study four out of 120 new-borns were positive for the virus.^6^ In a study among two hundred four deliveries, there were no positive newborns.^7^ In another two studies including 82 and 38 neonates born to COVID-19 positive mother also there were no positive cases.^8,9^ Systemic reviews have shown about three percent neonates acquired infection through possible vertical transmission.^10,11^ Though there are chances that neonates might have acquired COVID-19 after birth or positive reports may be due to contamination of samples, studies and case reports have shown COVID-19 virus to be present in amniotic fluid, cord blood, placenta and breast milk.^12-^ ^15^ The COVID-19 virus binds to Angiotensin-converting enzymes 2 receptor which is present in ovary, uterus, vagina, placenta, syncytiotrophoblast, cytotrophoblast, endothelium and vascular smooth muscles from primary and secondary villi. These findings point towards possible vertical transmission.

Most studies show higher caesarean rates among COVID-19 positive pregnancies. Forty-four to eighty percent deliveries were caesarean in different studies.^6,8,16,17^ This study shows a caesarean rate of sixty percent. Higher caesarean rate may be due to maternal altered food habits, physical activities and the stress related to the pandemic affecting the intrauterine growth in the weight of the baby.^18^ This study shows that risk of getting COVID-19 infection in vaginal delivery is 13.5 percent while in caesarean section it is 8.6 percent. A study from India shows 17.6 and 13.2 percent respectively.^5^ The study shows prematurity and low birth weight to be 12 and 14 percent respectively. A study by Sindy showed 38 percent case to be prematurity and 33 percent cases to be low birth weight.^19^ Another study shows that the most common adverse pregnancy outcome was premature.^7^ Need to terminate the pregnancy for maternal condition like pneumonia, acute respiratory distress syndrome, preeclampsia maybe the reason to have more premature deliveries in COVID-19 mothers. Though this study does not show significant higher prematurity and low birth weight babies.

A metanalysis showed among COVID-19 positive neonates fever was the most common neonatal symptom (40 %), followed by shortness of breath (28 %) and vomiting (24 %), while 20 % of neonates were totally asymptomatic.^17^ Our study also has similar result with fever in 40 percent cases among 10 COVID positive cases. Studies have shown that COVID-19 infected neonates have generally good outcome. Six of the 7 neonates were asymptomatic and 1 neonate needed respiratory support (indication being prematurity) which resolved after 48 hours.^2^ In another study where four (3.3%) of 120 new-borns were positive, all were asymptomatic.^6^ A report from India showed all six neonates to be asymptomatic.^20^ This study also has favourable outcome except in one which also had other severe comorbidity. Possible transfer of antibodies from mother to baby might have given protection against COVID-19 in newborns.^21^

The study shows the possibility of maternal to neonatal transmission. Although there is possibility that newborns can acquire COVID-19 infection following birth, there is still a high probability that it can be transmitted vertically. However, the outcome of these neonates who are infected is good. There are few limitations of this study. One of them is a small sample size. RT-PCR negative new-borns not routinely tested after 48 hours. Repeating test even after 48 hours could have even added the positive cases. Identifying the virus in amniotic fluid, cord blood and in the baby before twelve hours of life could have helped to establish the vertical transmission.

## CONCLUSIONS

COVID-19 in neonates is not an uncommon entity. There is a possibility of vertical transmission of coronavirus in neonates although the outcome is favourable. Further studies with larger sample size with testing of amniotic fluid, cord blood and placenta are required to validate the findings of our study.
